# Using physical contact heterogeneity and frequency to characterize dynamics of human exposure to nonhuman primate bodily fluids in central Africa

**DOI:** 10.1371/journal.pntd.0006976

**Published:** 2018-12-27

**Authors:** Victor Narat, Mamadou Kampo, Thibaut Heyer, Stephanie Rupp, Philippe Ambata, Richard Njouom, Tamara Giles-Vernick

**Affiliations:** 1 Institut Pasteur, Emerging Diseases Epidemiology Unit, Paris, France; 2 Eco-anthropologie et Ethnobiologie, CNRS/MNHN/Paris Diderot, France; 3 City University of New York, Lehman College, Department of Anthropology, New York, New York, United States of America; 4 Ministry of Agriculture and Rural Development, Yaoundé, Cameroon; 5 Centre Pasteur du Cameroun, Yaoundé, Cameroon; 6 Humans and the Microbiome Program, Canadian Institute for Advanced Studies, Toronto, Canada; Institute for Disease Modeling, UNITED STATES

## Abstract

Emerging infectious diseases of zoonotic origin constitute a recurrent threat to global health. Nonhuman primates (NHPs) occupy an important place in zoonotic spillovers (pathogenic transmissions from animals to humans), serving as reservoirs or amplifiers of multiple neglected tropical diseases, including viral hemorrhagic fevers and arboviruses, parasites and bacteria, as well as retroviruses (simian foamy virus, PTLV) that are pathogenic in human beings. Hunting and butchering studies in Africa characterize at-risk human social groups, but overlook critical factors of contact heterogeneity and frequency, NHP species differences, and meat processing practices. In southeastern Cameroon, a region with a history of zoonotic emergence and high risk of future spillovers, we conducted a novel mixed-method field study of human physical exposure to multiple NHP species, incorporating participant-based and ecological methodologies, and qualitative interviews (n = 25). We find frequent physical contact across adult human populations, greater physical contact with monkeys than apes, especially for meat handling practices, and positive correlation of human exposure with NHP species abundance and proximity to human settlement. These fine-grained results encourage reconsideration of the likely dynamics of human-NHP contact in past and future NTD emergence events. Multidisciplinary social science and ecological approaches should be mobilized to generate more effective human and animal surveillance and risk communications around neglected tropical diseases. At a moment when the WHO has included “Disease X”, a presumably zoonotic pathogen with pandemic potential, on its list of blueprint priority diseases as, new field-based tools for investigating zoonotic disease emergence, both known and unknown, are of critical importance.

## Author summary

Animal diseases that infect humans are a major threat to human health. Nonhuman primates’ genetic relatedness to human beings makes them an important source of disease spillovers into human populations. The central African rainforest has witnessed many infectious spillovers from primates, including multiple neglected tropical diseases. Previous studies have focused on hunting and butchering of primates as risky practices that expose central Africans to their diseases. These studies have not investigated how different kinds and frequencies of contact or the abundance of different monkey and great ape species affect these risks. We examined these factors in southeastern Cameroon, conducting a social sciences study of human physical contacts with nine different primate species. We found that Cameroonian adults had frequent physical contact with primates, and more with monkeys than apes, especially through handling meat for butchering, marketing, and preparation. People also had more contact with more abundant primate species living closer to villages. National and international authorities should support improved surveillance of humans and abundant monkey species, as well as popular messages to promote safe meat handling practices. Multidisciplinary social science and ecological approaches should be used to improve surveillance and communications with forest populations about neglected tropical diseases.

## Introduction

Zoonotic diseases constitute over 60% of emerging infectious diseases (EIDs), a major threat to global health [1–4]. Of these EIDs of zoonotic origin, 70% reportedly come from wild animals [5–6]. The World Health Organization (WHO) considers several zoonotic diseases from wild animals, including Ebola, Lassa, and Marburg, to pose major risks to global health because they are highly infectious, have had no licensed therapeutic or preventive measures, and elicit substantial fear [7–8]. Nonhuman primates (NHPs) occupy an important place in these transmissions, serving as reservoirs or amplifiers for pathogens, including several neglected tropical diseases (NTDs) that can infect human beings [8–13]. Nearly 45% of the pathogens shared by humans and NHPs are said to be “emerging” in humans, in the sense that they are appearing in humans for the first time or have recently increased [14–15].

Multiple factors, including genetic proximity between hosts, the adaptive ability of a pathogen, and human physical contact with NHPs and their bodily fluids are drivers of zoonotic transmissions [16–17]. Physical contact has constituted a particular focus for studies of zoonotic transmission, because certain past spillovers, including multiple hemorrhagic fevers, have emerged from human physical exposure to NHP biological fluids. Studies have identified several changes and associated human practices that facilitate this physical contact, including human demographic expansion, habitat encroachment and fragmentation, and the hunting and butchering of wild animals [18–21]. Animal factors can also influence physical contact, particularly species-specific ecologies, a species’ capacity to live near human settlement, and dietary modification from habitat disturbance [*eg* 22–24]. Understanding the complex interactions of human and animal factors that facilitate this physical contact and zoonotic transmission calls for a multi-disciplinary social sciences and ecological approaches [25–29].

Studies of hunting and butchering, notably in Africa where zoonotic emergences have reportedly occurred through these practices, seek to characterize the specific human populations with potential exposure to NHPs and their pathogens [18, 30–34]. These studies identify at-risk social groups and quantify potential exposure to NHP pathogens [30–31, 35].

Nevertheless, because of methodological limitations, these studies overlook critical factors of contact frequency and variability across types of human practice, contacts, and NHP species [36]. Such factors could yield more robust, specific identification of risky practices. Our systematic review of previous investigations of human-NHP contact found that these studies largely rely on one-time questionnaires, and sometimes on serological and/or fecal collections and mapping of spatial overlaps between people and NHPs [36]. One rare study that evaluated contact frequency with NHPs produced contact estimates only for NHP meat consumption, whereas another attempted to do so, but discarded its results [30, 37]. A more precise, longitudinal, granular investigation could evaluate cumulative frequencies of multiple practices over time. Studies of human-NHP contact have also neglected distinct, varied primate ecologies, including abundance and proximity to human settlements, which could potentially affect human-NHP contact frequency.

Because of methodological limitations, previous studies have also overlooked variability of several factors leading to physical exposure. Most questionnaire-based analyses, for instance, reduce multiple NHP species to broad categories of “monkeys” or “great apes” [30, 37, *cf* 35], without accounting for variable pathogen prevalence between primate species [37–39]. Critically, these studies treat practices like butchering as uniform and fixed, rather than varying by NHP species or changing over time [36]. Such evidence, not easily captured by quantitative data collection tools, provides invaluable insight into variability of physical exposures to NHPs. Finally, the marketing of wild meat, well studied in a conservation literature, may also constitute a zoonotic transmission risk [20–21, 40]. Relatively little is known about these risks, but the condition of marketed carcasses and meat varies, so that smoked and butchered meat will likely be less contagious than fresh, whole carcasses [40–43]. Who handles the carcass and meat under various conditions will affect risks of potential pathogenic transmission. Physical contact studies require mixed-method investigation to capture these critical factors, yield more precise evaluations of human contacts with diverse NHP species, and enhance surveillance to control zoonotic transmissions [44].

This novel study evaluates human-NHP contact frequency and variability on a granular level and in engagement with NHP species-specific ecologies in a central African region with a long history of zoonotic spillover. We conducted our mixed-method investigation in the dense forests of southeastern Cameroon, a region currently at high risk for hemorrhagic fever spillover and one in which pandemic HIV first emerged [45–46]. There, we assessed the frequency and variability of physical exposures to NHPs, critical factors affecting risk of zoonotic pathogen exposure. We moved beyond identification of at-risk populations to evaluate contact frequency with NHPs by contact type and gender; the influence of NHP ecologies on the frequency of human physical contact with NHPs; and the proportion and condition of NHP meat circulating in local and regional markets. Methods included a regional and longitudinal wild meat survey; a multi-village questionnaire; a 10-month, longitudinal participatory quantitative study among village inhabitants with daily data on their forest activities and NHP contacts; semi-structured individual and group interviews; participant-observations of forest-based activities; and a transect-based survey for an NHP census. We find that southeastern Cameroonians have variable frequencies of physical contact with NHPs, according to the type of contact and NHP species-specific ecologies. In comparison to great apes, physical contact with monkey species occurs more frequently and broadly across the population studied, particularly through butchering, marketing and meat preparation. Physical contact frequency varies significantly across monkey species, and this variability results from NHP species-specific relative abundance and proximity to human settlement. These results encourage reconsideration of the likely dynamics of human-NHP contact in past and future disease emergence events. Multidisciplinary social science and ecological approaches should be mobilized to generate more effective human and animal surveillance measures and risk communications around NTDs.

## Material and methods

### Study site

**Fig 1:**
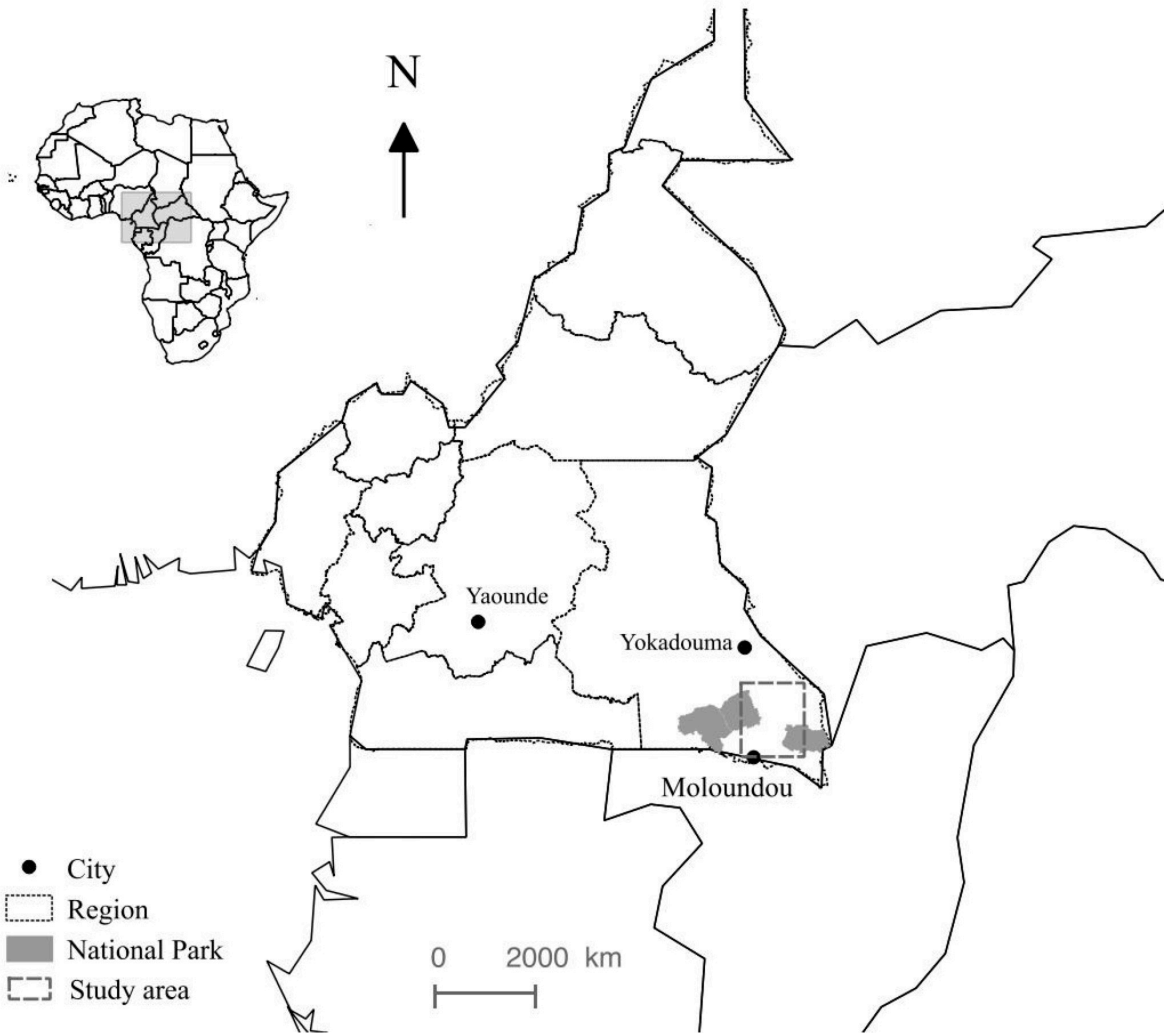
Location of the study. The map was developed with QGIS software v. 2.2.0 (https://qgis.org/fr/site/). Sources of layers: www.wri.org (cities and regions); www.protectedplanet.net (protected areas [48]).

We conducted the study in southeastern Cameroun, East Province, bordered by the Central African Republic, the Republic of Congo, and Gabon (Fig 1). This dense forest zone forms part of the Sangha river basin, a major tributary of the Congo River. Home predominantly to Baka and Bangando ethnic groups, as well as other ethnicities, southeastern Cameroon also contains nine NHP species including seven monkeys: *Cercopitecus nictitans*, *Cercopithecus cephus*, *Cercopithecus sclateri*, *Cercopithecus neglectus*, *Cercocebus agilis*, *Colobus guereza*, *Lophocebus albigena*, and two sympatric great apes—*Gorilla* and *Pan troglodytes*. Human populations depend heavily on the forest for commercial and subsistence agriculture, gathering, fishing and hunting. Commercial forest exploitation enterprises, primarily logging companies and hunting safaris, have operated in this region since the first decades of the twentieth century, whereas conservation efforts through collaborative efforts between the World Wildlife Fund (WWF) and the Ministry of Forests and Wildlife have been in effect for more than 20 years [45, 47]. “Eco-guards,” or armed forces for Ministry of Forests, have confiscated unregistered and automatic firearms in recent years. Throughout the region’s multi-use forest and in the Lobeke National Park (where activities other than seasonal gathering are prohibited), eco-guards now regularly conduct patrols to curtail hunting of all species and to prevent hunting of gorillas and chimpanzees.

Building on the long-standing relations between one anthropologist team member (SR) and local inhabitants, we conducted three extended field research trips in 2016 and 2017. We used multiple tools to collect quantitative and qualitative evidence.

### Participatory longitudinal quantitative methodology

We developed an original participatory quantitative data collection tool to evaluate physical contacts with NHPs at a granular level. In one target village, we recruited participants (“volunteers”) for a 10-month study (April 2016-January 2017). Inclusion criteria were any adult (>21 years-old), able to read and write, and residing in the study village. Each volunteer was trained to fill out a datasheet each day, documenting any NHP contact (injuries, hunting, selling/purchasing, butchering, cooking, eating) by species. VN trained and monitored volunteers’ data collection through two full days of participant-observations and frequent meetings with each participant. Of 22 volunteers recruited, 18 were included in the final dataset (8 women, 10 men). Three were excluded because they were unable to fill out datasheets, and one left the village after two months.

### Transect survey

In the same village, we ran transects to assess the abundance and proximity of the nine NHP species as an ecological index. From June 2016 to May 2017, six transects (each one km long) were conducted monthly to record signs of NHP presence including food remains, feces, nests, vocalizations and direct encounters. Transects were located perpendicular to the road bisecting the village at a distance of 1, 10 and 20 km from the village.

### Anthropological-historical investigation

We also performed 25 semi-structured oral historical interviews with individuals and small groups. We used snowball sampling, but sought to conduct interviews with men and women of different generational groups between 21 and over 90 years old, multiple ethnic groups, and in six different villages along the major regional road. These interviews offered insight into how local populations perceived NHP behavior, activities and mobility in forests and gardens, and how human-NHP relations and human practices around NHPs had altered over the previous 50 years. Interviews also yielded qualitative evidence about inhabitants’ understandings of the possibility of zoonotic disease transmission from NHPs. We conducted interviews in the Bangando or French languages and recorded them with participant authorization; interviews in Bangando language were translated into French, and all interviews transcribed. We also conducted lengthy participant-observations (n = 23, 116 hours) of farming, gathering, hunting, butchering, marketing, and wild meat preparation to evaluate these specific practices as physical contact with NHPs, as well as potential exposure to zoonotic disease. We took detailed notes of these activities.

### Multi-village questionnaire

To evaluate human activities and physical contacts with NHPs in the target and three neighboring villages along the major regional road, we conducted a questionnaire with 449 participants in May 2017. We developed our questionnaire after conducting preliminary analyses of our participatory quantitative tool, interviews, and participant-observations to elaborate a more accurate, locally appropriate data collection tool. In each village, we stratified the number of questionnaires with regional estimates of village populations. Sixteen local assistants conducted data collection by randomly selecting households and proposing the questionnaire to each adult present. Questionnaires gathered socio-demographic data, self-reported type of physical contact with each NHP species. We circumvented recall problems by asking subjects to estimate whether they had physical contact in the previous day, week, month, year, or more than one year. We coded data as the presence/absence of a specific NHP contact and as the estimated frequency. To do so, we calculated the inverse of the estimated number of days*100 (0 = never; 0.1 if >1 year ago; 0.3 within the past year; 3 within the past month; 14 within the past week, and 100 within the past day).

### Wild meat survey

We conducted a regional wild meat survey to track the proportion and condition of NHP carcasses compared to total wild meat offtake. To collect this data, we selected three informal market sites, because prohibitions on wild meat sales made it difficult to find wild meat at formal markets. To identify the range of hunted species, we selected one forest site outside of a village widely known for its hunting activities. A second site, 15 km away, was located at a crossroads frequented by logging trucks, whereas a third site, situated in a major regional town, reflected wild meat sales in an active market hub. From April 2016 to May 2017, three trained assistants monitored between one and five times per week the wild animal meat appearing in these markets. They noted species (vernacular name) and meat condition (whole, butchered pieces, cooked, fresh, or smoked). We employed an animal identification field guide to identify species’ scientific names [49].

### Data analyses

#### Analyses by gender of proportion of population exposed and mean frequency of contact

For each type of contact and each NHP species (including general categories “ape”, “monkey” and “all species), we calculated for men, women and in total, both the proportion of the population exposed at least once and the mean frequency of contact. We used the two independent datasets, one based on the open-ended responses of selected volunteers in our participatory longitudinal quantitative survey, the other on the larger sample assessed via the multi-village, closed-ended questionnaire. Differences in proportion of men and women involved at least once in a contact were tested with Chi2 test or Fisher exact test. We compared mean frequencies by gender and type of contact with the Wilcoxon test.

#### Influence of relative abundance index

We evaluated transect data using descriptive statistics to obtain the number of signs per species and village proximity (1km, 10km, and 20km) in an index of relative abundance (RAi), in which

$$RAi=\frac{Nb\;signs\;i\;at\;1\;km}{1}+\frac{Nb\;signs\;i\;at\;10\;km}{10}+\frac{Nb\;signs\;I\;at\;20\;km}{20}$$

This index allowed us to estimate species abundance according to distance from the target village. Two Spearman correlations were performed for each dataset to assess the influence of this relative abundance index on physical contact with each NHP species. The first correlation evaluated relative abundance and mean estimated frequencies of physical contact for each type of contact, and the second correlation assessed relative abundance and the proportion of population exposed at least once.

#### Descriptive analyses of wild meat data

We analyzed data from the wild meat survey by producing descriptive statistics to identify the proportion of great apes, monkeys, and specific NHP species of hunted animal species, as well as the state of the meat (fresh/smoked and whole/butchered). We also performed a Spearman correlation to assess the association between the proportion of wild meat for each NHP species and its relative abundance index.

#### Qualitative data analyses

We conducted qualitative content and discourse analysis of our anthropological-historical evidence to gain insight into current and past practices that brought people into physical contact with NHPs, as well as their understanding of the changing relations with and the mobilities and behaviors of NHPs [50]. After reading all transcriptions and notes, we conducted initial line-by-line inductive and deductive coding. We reorganized codes to synthesize and compare larger data segments, and then recoded our qualitative evidence to determine relations between data, codes and developing analytical categories. We developed our analysis of local interpretations of changing practices that brought them into physical contact with NHPs and the logics underpinning those practices. We subsequently triangulated results of all data collection tools to identify convergences and differences in practices, changes over time, and the local logics guiding these practices.

Independent coding and discussion of qualitative evidence, as well as continuous, long-term interaction with informants in southeastern Cameroon, ensured the high quality of our qualitative analyses.

### Ethical approvals

The Institut Pasteur Institutional Review Board (Decision No. 2014-30-IRB-01/02/03) and the Cameroon National Research Ethics Board for Human Health (Decision no. 2015/05/598/CE/CNERSH/SP) reviewed the protocol and informed consent forms and provided ethical approval for this study. We also received authorization to conduct the study from the Cameroon Ministry of Public Health. All participants, after receiving a written and oral description of the study and their rights, signed an informed consent form.

## Results

### Population exposed and contact frequency

Overall, our data show that physical contact with NHPs is high across the entire population, especially for meat handling, and that it is more frequent with monkeys than with great apes. Tables 1 and 2 summarize the proportion and frequency for each type of physical contact for monkeys, apes, and all species for our single-village participatory longitudinal quantitative survey dataset and for our multi-village questionnaire dataset. (See Supplementary File 1 for details by NHP species).

**Table 1:** Proportion of volunteers involved in physical contact and mean score^1^ of frequency by gender^2^ for type of contact for monkeys, apes and all species.

			Total (n = 18)	Women (n = 8)^1^	Men (n = 10)	p-value^2^
**Hunt**	All species	%	50^3^	13	80	*
		Mean (SD)	3.6 (9.8)	0.1 (0.2)	6.5 (12.7)	**
	Monkeys	%	50	13	80	*
		Mean (SD)	2.4 (5.6)	0.1 (0.2)	4.2 (7.2)	**
	Apes	%	0	0	0	/
		Mean (SD)	/	/	/	/
**Buy/Sell**	All species	%	100	100	100	NS
		Mean (SD)	20.2 (43)	34.2 (63.1)	9 (9.4)	NS
	Monkeys	%	94	88	100	NS
		Mean (SD)	10.3 (15.9)	13.3 (23.2)	7.9 (6.6)	NS
	Apes	%	44	38	50	NS
		Mean (SD)	0.9 (2)	1.1 (2.7)	0.8 (1.3)	NS
**Butcher**	All species	%	94	100	90	NS
		Mean (SD)	7.5 (10.9)	9.5 (12.7)	6 (9.6)	NS
	Monkeys	%	94	100	90	NS
		Mean (SD)	6.1 (7.3)	7.3 (7.9)	5.1 (7)	NS
	Apes	%	33	38	30	NS
		Mean (SD)	0.2 (0.5)	0.4 (0.7)	0.1 (0.2)	NS
**Cook**	All species	%	89	88	90	NS
		Mean (SD)	9 (12.4)	13.2 (17.6)	5.7 (4.5)	NS
	Monkeys	%	89	88	90	NS
		Mean (SD)	7.5 (8.1)	10.3 (11.3)	5.2 (3.6)	NS
		Mean (SD)	1.1 (2.5)	1.8 (3.7)	0.5 (0.5)	NS
	Apes	%	28	38	20	NS
		Mean (SD)	0.3 (0.9)	0.6 (1.4)	0.1 (0.1)	NS
**Consume**	All species	%	94	88	100	NS
		Mean (SD)	10.4 (12.9)	13.6 (17.6)	7.9 (7.6)	NS
	Monkeys	%	94	88	100	NS
		Mean (SD)	8.7 (8.7)	10.6 (11.3)	7.2 (6)	NS
	Apes	%	44	38	50	NS
		Mean (SD)	0.5 (1)	0.6 (1.4)	0.4 (0.7)	NS
**All physical**	All species	%	100	100	100	NS
		Mean (SD)	13.6 (14.3)	14.8 (17.1)	12.7 (12.5)	NS
	Monkeys	%	100	100	100	NS
		Mean (SD)	10.2 (8.9)	11.7 (10.9)	8.9 (7.3)	NS
	Apes	%	50	38	60	NS
		Mean (SD)	0.9 (1.8)	0.6 (1.4)	1.2 (2.1)	NS

1. Scores represent percentage of days with contact over a 10-month period

2. Comparisons of proportion of contact by gender group were performed by Fisher exact test, and those for frequencies with Wilcoxon test. NS: Not Significant. p-value < 0.05 (*); < 0.01 (**)

**Table 2:** Proportion of people in contact with NHP at least once and mean scores of estimated frequency^1^ (questionnaire data) by type of contact for monkeys, apes and all species.

Type of contact	Species		Total (n = 449)	Women (n = 203)	Men (n = 237)	p-value^2^
**Injury**	All species	%	6.9	2.5	10.5	**
		Mean (SD)	0.046 (0.676)	0.072 (0.983)	0.025 (0.199)	NS
	Monkeys	%	3.3	2.5	4.2	NS
		Mean (SD)	0.043 (0.681)	0.073 (0.988)	0.019 (0.199)	NS
	Apes	%	3.6	0	6.3	***
		Mean (SD)	0.004 (0.023)	0 (0)	0.007 (0.031)	***
**Hunt**	All species	%	49.2	10.8	81.4	***
		Mean (SD)	4.682 (16.977)	0.1 (1.004)	7.857 (20.984)	***
	Monkeys	%	49.2	10.8	81.4	***
		Mean (SD)	4.676 (16.978)	0.1 (1.004)	7.845 (20.987)	***
	Apes	%	22.7	1.5	40.1	***
		Mean (SD)	0.128 (0.781)	0.004 (0.036)	0.238 (1.064)	**
**Buy/Sell**	All species	%	79.5	75.4	84	*
		Mean (SD)	27.468 (39.518)	26.784 (39.568)	28.953 (39.991)	NS
	Monkeys	%	78.6	75.4	82.3	NS
		Mean (SD)	26.992 (39.276)	26.703 (39.61)	28.12 (39.526)	NS
	Apes	%	48.6	43.4	54.4	*
		Mean (SD)	1.5 (7.156)	0.835 (2.337)	2.126 (9.575)	0.06
**Butcher**	All species	%	90.0	85.7	93.7	**
		Mean (SD)	32.5 (41.4)	31.2 (40.1)	12.3 (26.5)	NS
	Monkeys	%	89.3	85.2	92.8	*
		Mean (SD)	32.533 (41.379)	31.213 (40.855)	34.167 (42.17)	NS
	Apes	%	69.5	59.6	78.1	***
		Mean (SD)	1.984 (7.394)	1.71 (7.589)	2.23 (7.323)	NS
**Cook**	All species	%	86.4	86.7	86.5	NS
		Mean (SD)	24.703 (36.884)	29.192 (39.373)	20.773 (34.102)	*
	Monkeys	%	83.7	85.7	82.3	NS
		Mean (SD)	24.654 (36.91)	29.122 (39.413)	20.741 (34.121)	*
	Apes	%	70.4	72.4	69.2	NS
		Mean (SD)	1.352 (3.191)	1.307 (3.087)	1.381 (3.23)	NS
**Consume**	All species	%	85.1	78.8	90.7	**
		Mean (SD)	31.1 (40.614)	28.912 (39.541)	33.834 (41.956)	NS
	Monkeys	%	84	78.3	89	**
		Mean (SD)	30.639 (40.414)	28.842 (39.58)	33.022 (41.603)	NS
	Apes	%	73.5	65.5	80.6	***
		Mean (SD)	2.033 (7.39)	1.253 (2.964)	2.716 (9.717)	*
**All physical**	All species	%	93.8	89.7	97.5	***
		Mean (SD)	42.533 (44.118)	37.715 (42.688)	46.807 (44.984)	NS
	Monkeys	%	93.3	89.2	97.1	***
		Mean (SD)	42.094 (44.034)	37.647 (42.737)	46.034 (44.853)	*
	Apes	%	82.9	76.9	88.2	NS
		Mean (SD)	3.044 (10.03)	2.226 (7.753)	3.798 (11.729)	NS

^1^ Estimated frequencies were calculated as follow: 0 = never. 0.1 = more than one year. 0.3 = during the previous year. 3 = during the previous month. 14 = during the previous week and 100 = yesterday.

^2^ Comparisons of proportion of contact by gender group were performed by Fisher exact test, and those for frequencies with Student t test. NS: Not Significant. p-value < 0.05 (*); <0.01 (**); < 0.001 (***). Differences in sample size between “Total” and “Women+Men” are due to 9 missing values for gender information.

#### NHP Injury

Injury from an NHP occurred rarely and involved few people. Our participatory longitudinal quantitative data yielded no injuries over the ten-month period, whereas 6.9% of multi-village questionnaire respondents did sustain injury. More men than women reported an NHP-related injury (10.5% and 2.5%, respectively). Our results show little difference in experiences of injury by a monkey (3.3%) or an ape (3.6%), but men were more likely than women to sustain injury from great apes (6.3% and 0%, respectively). Injuries from gorillas were most common (total 2.9%, 0% women, 5.1% men). Among the 31 people reporting an injury, one occurred in the previous week, one in the previous month, four in the previous year, and 25 prior to one year ago.

Injuries from gorillas, according to our qualitative interviews, could occur under different circumstances, in the context of hunting, but also that of gathering, cultivating forest gardens, and simply walking through the forest.

#### NHP hunting

Both multi-village questionnaire and single-village participatory longitudinal quantitative data reveal that about half the population hunted NHPs, primarily men (~ 80%) and to a lesser extent, women (~10%). Men also hunt NHPs significantly more frequently (about twice a month) than do women (about once every three years). Some 80% of men hunt monkeys once a month, whereas 40% hunt great apes once a year.

Participant-observations showed that hunting practices for NHPs varied considerably. Much hunting takes place in the early morning, particularly of gorillas pillaging forest gardens, but also at night with the use of flashlights. Many hunters used 12-caliber rifles, but others used crossbows and rarely, spears. Infrequently, gorillas and chimpanzees can be trapped in cable snares. Although hunters used dogs to track animals in general, we did not observe dogs employed for hunting NHPs.

Hunting techniques had reportedly changed somewhat over time, according to some informants. As one longtime hunter observed, “Our grandparents would kill the chimpanzee with a crossbow and poisoned arrows. They would do the same for gorillas that were in the trees. On the ground, they might use a spear, but it’s difficult to kill chimpanzees with a spear.” Rifles had largely replaced these older weapons.

Our participant-observations and qualitative interviews revealed that monkey hunting does not necessarily lead to high risk of exposure to NHP pathogens. In our observations, we found hunters would simply carry the dead monkey by the tail or in a bag, and would sell the carcass fresh and whole, unless they chose to consume the animal themselves [51].

Qualitative interviews also indicate that our informants generally agreed that great ape and monkey hunting had declined in recent decades. Multiple informants recalled that hunting intensified in the last decades of the twentieth century with expanded access to firearms, but had declined over the last twenty years with the intensification of anti-poaching patrols and especially arms confiscations in recent years. “Before the park,” one village inhabitant reflected, “everyone could go into the forest and work without a problem. But now, you’re always worried because the agents (eco-guards) can come. Even setting small traps is difficult.”

#### NHP marketing

Our data show widespread participation in marketing (defined as selling or purchasing) NHP meat on village and multi-village scales. Most multi-village questionnaire respondents marketed NHP meat (79%, with 79% implicated in marketing of monkeys, and 49% for apes); data similarly show all 18 volunteers in our participatory longitudinal survey involved at least once in NHP marketing (94% for monkeys and 44% for apes). Although men engaged in marketing NHPs significantly more than women, we found at the species level no statistical difference of frequency of marketing NHPs, except for chimpanzee and colobus, for which men had higher scores. On average, marketing occurred once every 4 to 5 days in the overall population.

#### NHP butchering

Butchering NHPs is a widespread, frequent event. Over their lives, 90% of questionnaire respondents butchered NHPs (89% monkeys, 69% apes). During the study period, 94% of volunteers butchered an NHP (94% monkeys, 33% apes). Questionnaire and participatory data revealed that butchering NHPs occurred twice a week to twice a month, and that more men were involved in butchering NHPs but with no difference in frequency of butchering of all NHPs (except for chimpanzees, for which questionnaire data showed that men butchered more frequently).

Qualitative data revealed differences between monkey and great ape butchering, as well as changes in great ape butchering practices over the last two decades. Interviews and participant-observations revealed that monkey and great ape butchering could involve multiple actors and procedures (See Supplementary File S1 Movie for an example). Butchering in this setting was not simply a practice, but a genuine skill honed over years of experience. One woman observed, “To cut meat, I learned at home from my father, but my husband really developed my butchering skills because we were in the forest together a lot.”

Women or men butchering a dead monkey might acquire it whole, expose the carcass directly to fire in order to singe the fur and partly coagulate the blood, and then gut and cut up the remaining meat into pieces with sharp knives. At other times, a hunter (usually male) might return home, prepare the entrails to share with other men, and then hand over rest of the monkey carcass to female family members to cut and prepare. In both cases, exposure to monkey bodily fluids appears widely distributed across women and men.

The intensification of conservation measures in the previous two decades has influenced great ape butchering. Whereas the killing of a gorilla, particularly one that ravaged fields, once occasioned celebrations in the village, these public festivities have now entirely disappeared. As one aging hunter noted, “These days, with ‘dobi dobi’ (WWF), you have to kill and eat these animals in secret.” Hunters now skin and butcher gorillas, for instance, under the cover of the forest, smoke its meat, and carry smaller meat, deboned pieces home to consume or sell. Such measures render the meat more portable and less easily identifiable as gorilla, thus protecting the hunter from anti-poaching patrols.

Previously, hunters would carry a killed gorilla home to show to children and to instruct them in appropriate behaviors when encountering a live gorilla in the forest. Family members, both women and men, would then butcher the carcass. As a result of conservation measures, potential exposure to gorilla bodily fluids now appears more concentrated on hunters themselves than in prior decades, when more participants butchered the animal.

#### NHP cooking

Both women and men are involved in cooking NHPs. Some 86% of our multi-village questionnaire respondents had cooked an NHP (84% monkey, 70% ape), although male respondents frequently added that cooking was “for women”. Cooking contact occurred on average every 10 days for single-village volunteers, and every four for multi-village questionnaire respondents. We found no gender differences in the proportion of the population involved and in frequency of cooking among volunteers, although the multi-village questionnaire data showed that women cooked only one species, *C*. *nictitans*, significantly more frequently than men.

Our participant-observations shed light on the disparities between male perceptions of cooking as a female activity and the non-gendered practices of NHP preparation, since hunters spending several days in the forest would frequently prepare and consume the entrails of killed monkeys.

#### NHP consumption

NHP consumption is also widespread. Some 85% of questionnaire respondents ate NHP meat at least once in their lifetimes (84% monkeys, 73% apes), and 94% of volunteers did so over the course of the study (94% monkeys, 44% apes). Our multi-village questionnaire analyses revealed that men consumed more NHPs, and significantly more gorilla and *Cercopithicus cephus* (moustached guenon), than women. Single-village participatory survey volunteers, however, displayed no difference between male and female consumption of NHPs, except for one monkey species (*Cercocebus agilis*). NHP consumption frequency estimates ranges from once every 10 days (volunteers) to once every three days (multi-village questionnaire).

### Variability in the condition of marketed wild meat and exposure to monkeys and apes

**Fig 2:**
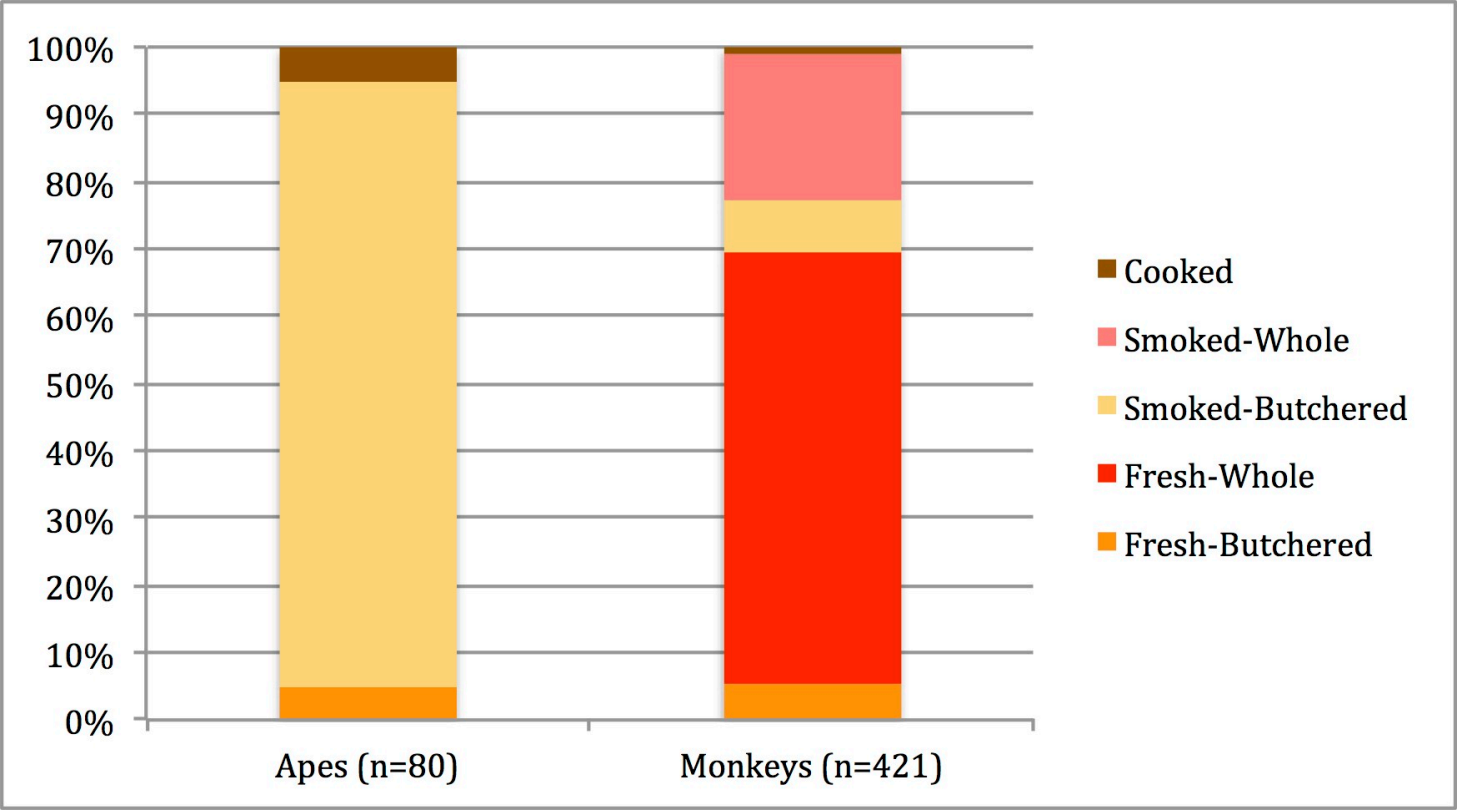
Proportion of the condition of great ape and monkey meat sold in wild meat markets.

Analyses of our regional wild meat survey indicated that great ape meat represented 3% and monkeys 16% of offtake (n = 2592). However, monkeys tended to be sold fresh and whole (64%), whereas great apes were marketed smoked and butchered (90%) (Fig 2). Purchasers likely faced greater NHP bodily fluid exposure from fresh, whole monkey carcasses than from that of great ape meat. Table 3 summarizes results at the species level.

**Table 3:** Occurrence and proportion of NHP species in wild meat survey.

	Total	Fresh/smoked meat			Whole/Cut/Cooked			
		Fresh	Smoked	NA^3^	Whole	Cut	Cooked	NA
No. Unidentified primates (%)^1^	20 (0.8)	10 (50.0)	7 (35.0)	3 (15.0)	16 (80.0)	2 (10.0)	0	2 (10.0)
**No. Monkeys (%)**	**421 (16.2)**	**289 (68.6)**	**122 (29.0)**	**10 (2.4)**	**358 (85.0)**	**54 (12.8)**	**5 (1.2)**	**4 (1.0)**
*Cercocebus agilis*	92 (3.6)	58 (63.0)	32 (34.8)	2 (2.2)	74 (80.4)	14 (15.2)	3 (3.3)	1 (1.1)
*Cercopithecus cephus*	80 (3.1)	50 (62.5)	29 (36.3)	1 (1.3)	71 (88.8)	7 (8.8)	1 (1.3)	1 (1.3)
*Cercopithecus neglectus*	18 (0.7)	15 (83.3)	3 (16.7)	0	17 (94.4)	1 (5.6)	0	0
*Cercopithecus nictitans*	68 (2.6)	54 (79.4)	10 (14.7)	4 (5.9)	53 (77.9)	14 (20.6)	0	1 (1.5)
*Cercopithecus sclateri*	46 (1.8)	43 (93.5)	2 (4.3)	1 (2.2)	37 (80.4)	7 (15.2)	1 (2.2)	1 (2.2)
*Colobus guereza*	62 (2.4)	42 (67.7)	20 (32.3)	0	55 (88.7)	7 (11.3)	0	0
*Lophocebus albigena*	55 (2.1)	27 (49.1)	26 (47.3)	2 (3.6)	51 (92.7)	4 (7.3)	0	0
**No. Apes (%)**	**80 (3.1)**	**6 (7.5)**	**74 (92.5)**	**0**	**0**	**76 (95.0)**	**4 (5.0)**	**0**
*Gorilla*	57 (2.2)	5 (8.8)	52 (91.2)	0	0	53 (93.0)	4 (7.0)	0
*Pan troglodytes*	23 (0.9)	1 (4.3)	22 (95.7)	0	0	23 (100.0)	0	0
**TOTAL**^**2**^	**521 (20.1)**	**305 (58.5)**	**203 (39.0)**	**13 (2.5)**	**374 (71.8)**	**132 (25.3)**	**9 (1.7)**	**6 (1.2)**

1. Percentages were calculated by species as a proportion of total number of occurrences and by condition (fresh/smoked and whole/cut/cooked).

2. Based on 2592 carcasses recorded.

3. NA: missing values

### NHP ecologies and physical contact

**Fig 3:**
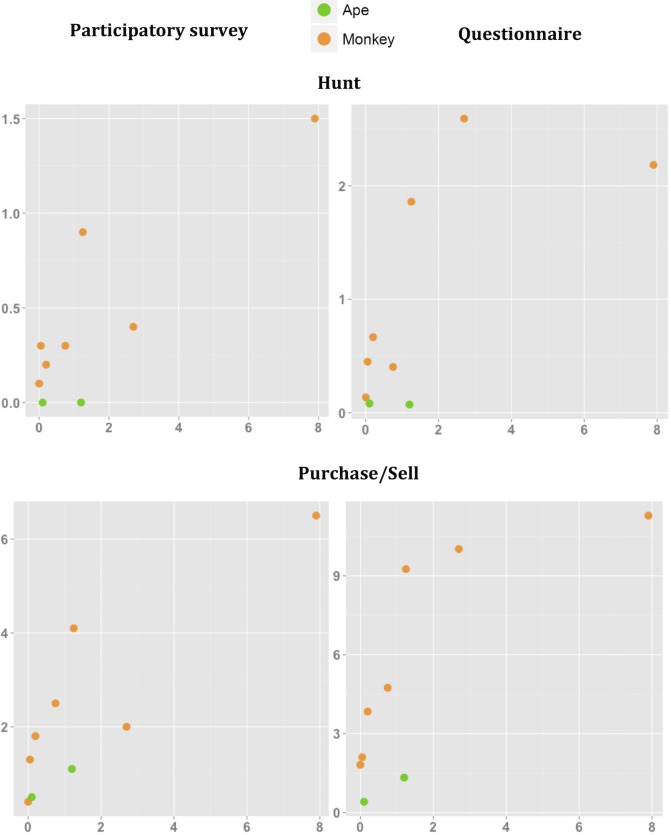
Mean frequencies (*100) of physical contact by physical contact via Hunt and Purchase/Sell, and NHP relative abundance index. Means from two independent datasets collected through a participatory longitudinal survey and questionnaire are shown.

**Fig 4:**
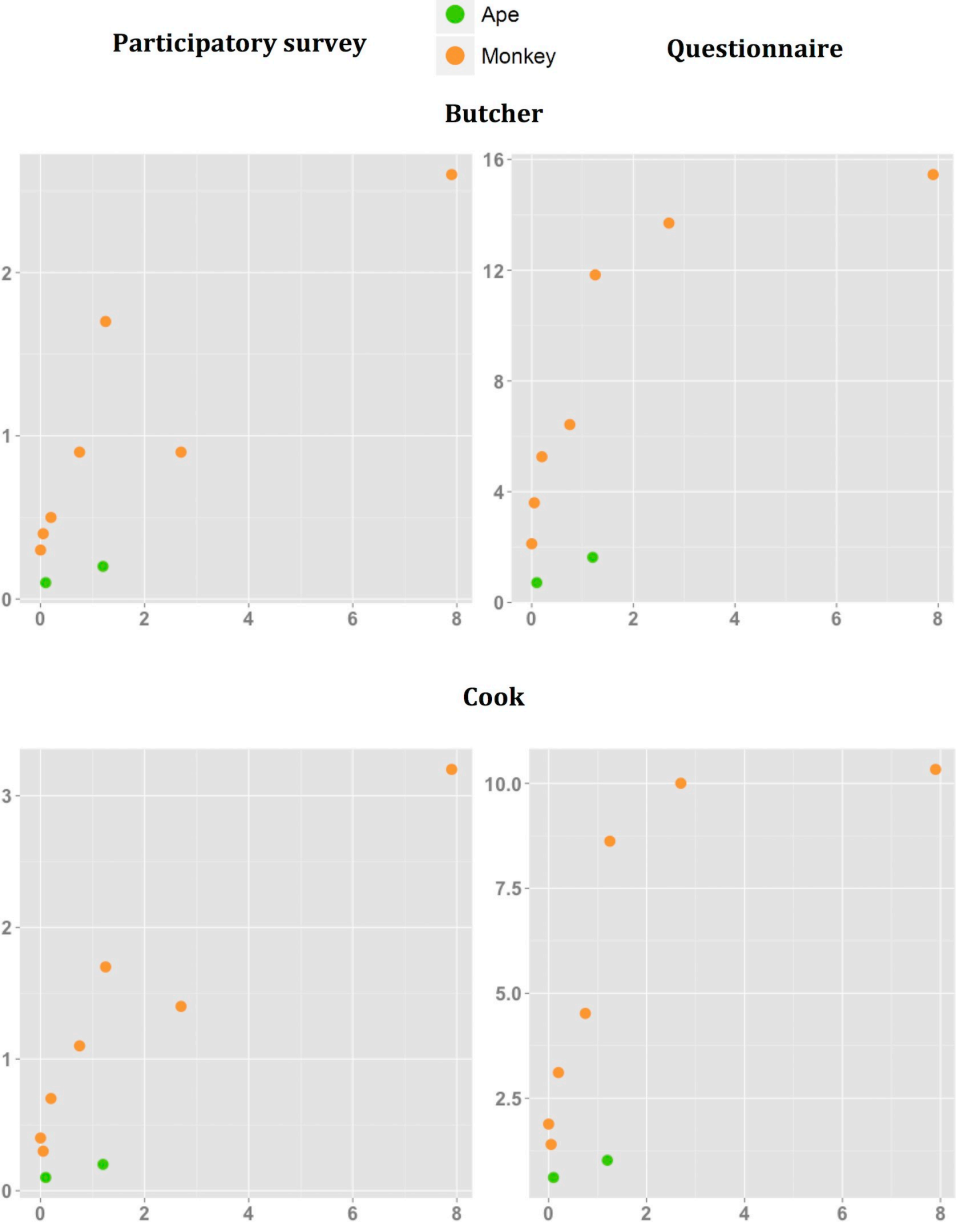
Mean frequencies (*100) of physical contact by physical contact via Butcher and Cook, and NHP relative abundance index. Means from two independent datasets collected through a participatory longitudinal survey and questionnaire are shown.

**Fig 5:**
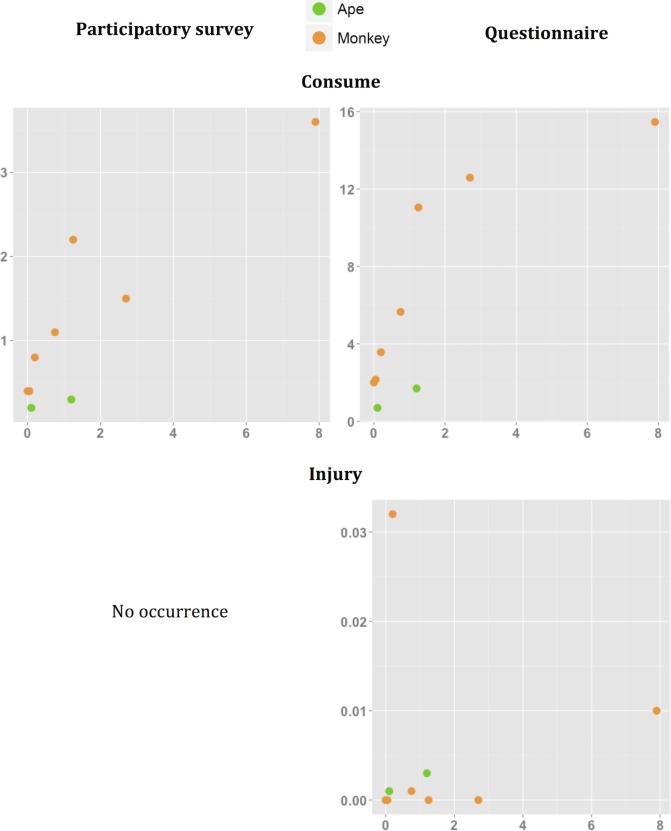
Mean frequencies (*100) of physical contact by physical contact via Consume and Injury, and NHP relative abundance index. Means from two independent datasets collected through a participatory longitudinal survey and questionnaire are shown.

People reported more physical contact with certain NHP species. Responses to our multi-village questionnaire and single-village participatory quantitative tool yielded a highly positive, significant correlation between estimated frequencies of almost all types of physical contact with NHP species. The sole exceptions here were for injury and NHP relative abundance index (Table 4, Fig 3, Fig 4, Fig 5). Similarly, the proportion of population exposed was also positively correlated with the relative abundance index except for injury and butchering, as measured by the multi-village questionnaire (Table 5, Fig 3, Fig 4, Fig 5). Table 6 summarizes the relative abundance index for each NHP species. The wild meat survey revealed that the correlation between the proportion of primate species (as marketed meat) and the relative abundance index was not significant. Great ape species tended to have lower contact scores according to their relative abundance index (Fig 3, Fig 4, Fig 5). The Spearman coefficient was higher when the correlation was performed for only monkeys, rather than for all NHP species (Table 4, Table 5). This result suggests that all physical contact with great apes except for injury is less frequent than expected from their relative abundance index.

**Table 4:** Spearman tests for each type of contact between estimated frequency of contact and relative abundance.

	Type of contact	Participatory survey data		Questionnaire data	
		Rho	p-value	Rho	p-value
**Monkeys only**	Injury	**/**	**/**	0.24	0.6
	Hunt	0.90	**	0.86	*
	Buy/Sell	0.89	**	1	***
	Butcher	0.94	**	1	***
	Cook	0.93	**	0.96	**
	Consume	0.95	***	1	***
	All physical	0.96	**	1	***
**All species**	Injury	**/**	**/**	0.24	NS
	Hunt	0.66	0.06	0.62	0.09
	Buy/Sell	0.8	**	0.75	*
	Butcher	0.71	*	0.75	*
	Cook	0.72	*	0.73	*
	Consume	0.73	*	0.75	*
	All physical	0.85	**	0.75	*

For estimated frequency, correlations are shown for the participatory longitudinal survey and questionnaire data. Relative abundance of NHP species was calculated from the transect survey by dividing the number of signs of a species by distance from the village. NS: Not Significant. p-value < 0.05 (*); <0.01 (**); < 0.001 (***).

**Table 5:** Spearman tests for each type of contact between proportion of the population exposed at least once and relative abundance.

	Type of contact	Participatory survey data		Questionnaire data	
		Rho	p-value	Rho	p-value
**Monkeys only**	Injury	**/**	**/**	0.64	NS
	Hunt	0.95	***	0.89	**
	Buy/Sell	0.94	**	1	***
	Butcher	0.96	**	0.56	NS
	Cook	0.96	**	1	***
	Consume	0.95	***	1	***
	All physical	0.93	**	1	***
**All species**	Injury	**/**	**/**	0.43	NS
	Hunt	0.70	*	0.73	*
	Buy/Sell	0.90	***	0.93	***
	Butcher	0.78	**	0.56	NS
	Cook	0.73	*	0.98	***
	Consume	0.81	**	0.98	***
	All physical	0.82	**	0.98	***

For proportion, correlations are shown for the participatory longitudinal survey and questionnaire data. Relative abundance of NHP species was calculated from the transect survey by dividing the number of signs of a species by distance from the village. NS: Not Significant. p-value < 0.05 (*); <0.01 (**); < 0.001 (***).

**Table 6:** Total number of signs of presence recorded on transects (conducted monthly, June 2016 to May 2017) according to distance from the village and NHP species.

	Total	1 km	10 km	20 km	Relative Abundance^1^
**Monkeys**	**80**	**8**	**25**	**47**	**12.85**
*Cercocebus agilis*	2	0	2	0	0.2
*Cercopithecus cephus*	11	2	5	4	2.7
*Cercopithecus neglectus*	0	0	0	0	0
*Cercopithecus nictitans*	48	5	15	28	7.9
*Cercopithecus sclateri*	4	1	2	1	1.25
*Colobus guereza*	1	0	0	1	0.05
*Lophocebus albigena*	14	0	1	13	0.75
**Apes**	**25**	**0**	**1**	**24**	**1.3**
*Gorilla gorilla*	23	0	1	22	1.2
*Pan troglodytes*	2	0	0	2	0.1

^1^Relative abundance (RA) is calculated for the species *i* as follow:
$$RAi=\frac{Nb\;signs\;i\;at\;1\;km}{1}+\frac{Nb\;signs\;i\;at\;10\;km}{10}+\frac{Nb\;signs\;I\;at\;20\;km}{20}$$

Our qualitative evidence also reveals considerable variation in human perceptions of different NHP species, which may also affect hunting practices and physical contact more generally beyond the relative abundance index, consumption preferences, or conservation policies. Although mythical tales (still recounted in this region) portray gorillas and chimpanzees as similarly gluttonous and antisocial, 12 recollections of encounters depict gorillas as highly aggressive and dangerous, albeit with notable exceptions, and chimpanzees as more calculating in their behaviors, pleading for their lives when confronted by hunters. One longtime hunter admitted, “To kill a chimpanzee, it isn’t easy, because it acts like a human. When you find one in a trap, to kill it you have to be brave. When you raise your spear, it’ll raise its hands and go, ‘Aie, aie aie!’ (pleading). It doesn’t make you happy…” Another hunter claimed, “I have never killed a chimp. They are too much like people.” In four interviews, informants also portrayed the black and white colobus (*Colobus guereza)* as disconcertingly “human” in its habits, consuming salt and sleeping in beds that it finds in human forest dwellings, or even using occult forces to escape human hunters who try to shoot it down. Although difficult to show conclusively that these perceptions shape hunting patterns, they suggest a reticence to hunt these species.

## Discussion

Physical exposure to NHP bodily fluids is a risk factor that can facilitate pathogenic spillover and potentially result in the emergence of NTDs and other diseases. Our novel study evaluated human-NHP contact frequency and heterogeneity on a granular level and in engagement with NHP species-specific ecologies. We integrate quantitative granular geographical, activity, social, and transect data with qualitative anthropological, historical evidence to characterize contact patterns between people and nine NHP species in southeastern Cameroon, a region where pandemic HIV-1M first emerged. We find frequent, broad physical contact across adult populations, greater physical contact with monkeys than apes, especially for meat handling practices, and increased human exposure and physical contact with higher NHP species abundance and proximity to human settlement. These fine-grained results encourage reconsideration of the likely dynamics of human-NHP contact in past and future disease emergence events, highlighting the contributions of meat handling and NHP-specific ecologies to human physical exposures.

### All southeastern Cameroonian populations studied have frequent physical contacts with NHPs, especially through meat handling

Whereas previous studies of physical contact with NHPs identify target populations at risk [30, 37] our longitudinal study also measured the frequency of physical contacts. We found that all southeastern Cameroonian adults studied have frequent physical contact with NHPs. Indeed, analyses of contact frequency showed more heterogeneous, less gendered physical exposures to NHPs than previously indicated. Nearly 11% of women questionnaire respondents having hunted a monkey at least once, and more men engaged in butchering than some studies have previously reported [30, 37 but see 35]. Marketing, butchering, cooking and eating NHP meat occurred very frequently, with approximately 85% of the population involved at frequencies of once to twice weekly.

Hunting engaged about 50% of the overall population, primarily men, occurring approximately once a month. That said, hunting itself does not lead to high exposure contact with NHP bodily fluids. Rather, NHP attacks during hunting or other activities (injury) and butchering injuries will facilitate physical contact with NHP bodily fluids [52–53]. Our analyses showed that injury was the least widespread (6.9%) and least frequent contact, occurring just once every several years.

Generally, these results suggest that evaluations of at-risk sub-groups may be of limited use in regions where zoonotic transmission risk likely depends more on exposure frequency. More useful would be for surveillance and risk communications efforts to target the widespread and frequent meat handling and preparation practices [20, 35, 38]. To obtain frequency data, field investigations should also implement new methodologies tools beyond questionnaires to assess frequency.

### The frequencies and proportions of human populations sustaining NHP physical contact vary according to NHP type (monkey or ape)

In line with prior studies, southeastern Cameroonian participants report more physical contact with monkeys than with great apes [30, 37]. In this study, people have 15 times more frequent physical contact with monkeys than with great apes, except for injury. That said, over a third of male multi-village questionnaire respondents report having hunted great apes in their lifetimes, exceeding previous reports in Cameroon (10% in [30]). Our higher reported physical contacts with great apes may reflect changing hunting patterns over time, regional differences in practices or NHP densities, effectiveness of great ape conservation efforts in the region, or possibly our research team’s long-term presence in the region, which cultivated local population trust and led to more reliable data.

We identify frequency and heterogeneity in meat handling practices for great apes and monkeys, which can differentially affect population exposure to NHP bodily fluids. Monkeys tend to be transported, marketed, and purchased whole, leading to little or no bodily fluid exposure for hunters, but elevated and more frequent exposure for NHP meat marketers and preparers. Although physical contact with great apes is less elevated and less frequent, this risk was concentrated in hunters. Hunters butcher and smoke great ape meat in the forest before marketing, so that they will likely sustain higher NHP bodily fluid exposure than other social groups. Because a broad segment of the population frequently engages in monkey meat handling, our findings suggest that zoonotic disease prevention measures could promulgate locally adapted, acceptable safety practices around meat handling.

### NHP species-specific ecologies also affect human physical contact

NHP species-specific ecologies also affect human physical contact: higher species abundance and proximity to human settlement is strongly associated with increased human exposure and physical contact frequency, except for injury. Human-NHP physical contacts are not just a consequence of heterogeneous human practices and preferences, but also those of NHP species. To our knowledge, this is the first time that such data have been integrated into physical contact analyses.

This finding has significant implications for human-NHP physical contact. Attention to frequency and type of contact with each species is crucial, because disease prevalence differs across NHP species, especially for simian retroviruses, intestinal helminths and bacteria [38–39, 54]. Certain NHP species ecologies may reflect greater tolerance for human presence, among other factors. A comparison of our findings linking NHP species-specific ecologies and estimated frequencies of physical contacts with those in other sites in central Africa would be useful, particularly when NHP species-specific abundance data are increasingly available because of threats to these species [55]. In light of these results, surveillance and monitoring of abundant NHP species in close proximity to human settlement should be considered.

The one exception to this finding was NHP-inflicted injury, particularly by great apes. Gorillas, most implicated in human injury, can attack without hunting provocation, inflict greater damage than monkeys, and constitute an important reservoir for SFV and STLV retroviruses [32–33, 52, 56–60]. Our analysis suggests physical contact through injury is relatively infrequent and does not follow the same pattern as more frequently practiced hunting, butchering or meat handling. We found no correlation between frequency of injuries and NHP relative abundance and human settlement. This finding may be related to species-specific behavior toward human beings. The finding is also consistent with our historical research on HIV-1M emergence, which suggests that beyond (chimpanzee) hunting, other pathways leading to spillover into humans should be considered [45]. In addition, it can also contribute to characterizations of the dynamics of viral hemorrhagic fever spillovers into central African forest inhabitants.

### Mixed methods yield better insight into frequency and heterogeneity of human practices and NHP ecologies

Our mixed-method approach southeastern Cameroon is part of a small, emerging field that mobilizes anthropological and ecological investigation to understand more fully the processes and practices potentially leading to zoonotic transmission. More than a decade ago, Augustin Fuentes investigated in Gibraltar and Bali bite rates that human beings sustained in their interactions with macaques and the potential for zoonotic transmission. His study showed that multiple factors shaped pathogenic transmission risk, including macaques’ species-specific behaviors and geographies, as well as human “demographic, cultural and contact characteristics.” [61]. More recently, Bonwitt and colleagues have productively used mixed methods to document direct and indirect contacts between people and rats, which contribute to the transmission of Lassa fever [26]. Leach and colleagues conducted a comparative analysis of zoonotic disease transmission in Africa, contending that ecological changes and diverse human social engagements with them not only influence pathogenic transmission, but also shape which social groups are most exposed [62].

Our mixed-method approach had several advantages that improve upon exclusively questionnaire-based studies. The participatory quantitative survey provided more granular, longitudinal data than can be collected through questionnaires, and it yielded precise assessment of human physical contact frequency with NHPs. Collecting data on species-specific relative abundance and proximity to human settlement, and putting these data into dialogue with questionnaire and participatory longitudinal survey data permitted us to evaluate how specific NHP ecological factors influenced physical contacts. Our participant-observations and interviews provided rich evidence concerning local observations and perceptions of different NHP species, of reticence or willingness to hunt various species, and of heterogeneous practices that changed over time but for which we had no other sources documenting these changes. This qualitative research enabled us to develop a more precise, relevant questionnaire that more accurately captured human activities, NHP contacts, and their frequencies. Future studies using questionnaires should include the estimated frequencies of physical contact. Most important, these methods revealed that Southeastern Cameroonians have many types of engagements with NHPs and that they attribute wide-ranging meanings to these animals, as meat, sources of danger and pillage, and as “nearly human.” These insights too help us to understand why, how, and how frequently people engage in physical contacts with NHPs.

In a region with active biodiversity conservation and NHP protection, this study also cultivated local population trust because of one author’s (SR) long-term research experiences and her established independence from conservation efforts. This trust was of primordial importance: because local populations knew that our study was independent from conservation efforts and that they would not be arrested for engaging in potentially illegal practices, they gave us access to rich data on an unofficial wild meat trade and divulged illegal activities in all data collection tools.

Our mixed methods approach also had some drawbacks. We limited the number of inclusions in our participatory study, because volunteer training was time-consuming and daily reporting generated a high volume of data. Our recruitment for this tool was also biased to favor literate participants. Frequent hunters may also have feared reporting their game offtake, although questionnaires also suffer this bias. Great ape hunting was not recorded during the ten-month participatory survey. Finally, due to study limitations, we do not have prevalence estimates of NHP-transmitted viral infections in human beings. It would be useful to replicate the participatory survey elsewhere in Africa to estimate NHP contact frequency and to conduct seroprevalence studies with the same participants.

Several factors counterbalance these shortcomings. The detail of daily participant data compensates for small number of volunteers, and we have relatively high confidence in the accuracy of data reported. One volunteer, for instance, reported hunting almost every two days. This high hunting frequency exceeded our multi-village questionnaire results. Although the absence of reporting of great ape hunting may have resulted from volunteer reticence to divulge this illegal practice, two months after the end of the participatory survey, we learned that one volunteer killed a gorilla near his field because it damaged his crops. Additionally, volunteers reported other physical contacts with great apes through butchering, cooking and eating. We therefore are confident in the robustness of this data.

New field-based tools for investigating zoonotic NTD emergence, both known and unknown, are of critical importance now. In February 2018, the WHO included “Disease X” on its list of blueprint priority diseases, prioritizing this unknown pathogen for research and development because of its potential risk to cause a public health emergency, and because few or no countermeasures exist [7]. Predicting how, where, or when Disease X might occur will require multiple methods, including pathogen discovery investigation in animal and human populations. Rigorous multidisciplinary tools also have a significant place here. They can document heterogeneous practices, contacts, and animal ecologies on the frontiers of new zoonotic emergences and contribute to more targeted, effective surveillance and risk communications.

## Supporting information

S1 Table Supporting information table. (DOCX)

S1 Movie Supporting information movie. **Hunting and butchering a monkey (*Cercopithecus nictitans*).** This movie was filmed during a participant-observation in May 2016. VN followed one hunter conducting a monkey hunt near a forest camp between 6 to 10 AM. This movie shows the shooting, transport and butchering steps. During this observation, two monkeys were killed, but only one shooting and butchering was filmed. This movie illustrates one example of hunting and butchering, but does not reflect the diversity of practices.
(M4V)
