# Supplementary material for: Using physical contact heterogeneity and frequency to characterize dynamics of human exposure to nonhuman primate bodily fluids in central Africa
Source: PLoS Negl Trop Dis. 2018 Dec 27;12(12):e0006976. doi: 10.1371/journal.pntd.0006976 (PMC6307716; doi:10.1371/journal.pntd.0006976)
Supplement: S1 Table — (DOCX) [file pntd.0006976.s001.docx]

**Supporting information 1**

**S1_A: Proportion of volunteers involved in physical contact and mean score^1^ of frequency by gender^2^ for type of contact and NHP species. Results for monkeys, apes and all species are in bold.**

|  |  |  | **Total (n=18)** | **Women (n=8)^1^** | **Men (n=10)** | **p-value^2^** |
| --- | --- | --- | --- | --- | --- | --- |
| **Hunt** | *Cercocebus agilis* | % | 22 | 0 | 40 | NS |
|  |  | Mean (SD) | 0.2 (0.3) | 0 (0) | 0.3 (0.4) | 0.05 |
|  | *Cercopithecus cephus* | % | 39 | 13 | 60 | 0.07 |
|  |  | Mean (SD) | 0.4 (1) | 0 (0.1) | 0.8 (1.3) | * |
|  | *Cercopithecus neglectus* | % | 17 | 0 | 30 | NS |
|  |  | Mean (SD) | 0.1 (0.3) | 0 (0) | 0.2 (0.4) | NS |
|  | *Cercopithecus nictitans* | % | 33 | 13 | 50 | NS |
|  |  | Mean (SD) | 1.5 (4.5) | 0 (0.1) | 2.6 (5.9) | 0.07 |
|  | *Cercopithecus sclateri* | % | 28 | 0 | 50 | * |
|  |  | Mean (SD) | 0.9 (2.9) | 0 (0) | 1.6 (3.8) | * |
|  | *Colobus guereza* | % | 17 | 0 | 30 | NS |
|  |  | Mean (SD) | 0.3 (0.9) | 0 (0) | 0.5 (1.2) | NS |
|  | *Lophocebus albignea* | % | 22 | 0 | 40 | NS |
|  |  | Mean (SD) | 0.3 (0.5) | 0 (0) | 0.5 (0.7) | 0.06 |
|  | *Gorilla gorilla* | % | 0 | 0 | 0 | / |
|  |  | Mean (SD) | / | / | / | / |
|  | *Pan troglodytes* | % | 0 | 0 | 0 | / |
|  |  | Mean (SD) | / | / | / | / |
| **Buy/Sell** | *Cercocebus agilis* | % | 61 | 50 | 70 | NS |
|  |  | Mean (SD) | 1.8 (4.2) | 3.1 (6.3) | 0.7 (0.8) | NS |
|  | *Cercopithecus cephus* | % | 78 | 88 | 70 | NS |
|  |  | Mean (SD) | 2 (4.3) | 3.5 (6.3) | 0.9 (0.8) | NS |
|  | *Cercopithecus neglectus* | % | 22 | 25 | 20 | NS |
|  |  | Mean (SD) | 0.4 (1.2) | 0.7 (1.8) | 0.1 (0.3) | NS |
|  | *Cercopithecus nictitans* | % | 100 | 100 | 100 | NS |
|  |  | Mean (SD) | 6.5 (13.1) | 10.5 (18.9) | 3.3 (4.7) | NS |
|  | *Cercopithecus sclateri* | % | 100 | 100 | 100 | NS |
|  |  | Mean (SD) | 4.1 (9) | 6.8 (13.2) | 2 (2.3) | NS |
|  | *Colobus guereza* | % | 39 | 38 | 40 | NS |
|  |  | Mean (SD) | 1.3 (3.2) | 2.1 (4.5) | 0.6 (1.5) | NS |
|  | *Lophocebus albignea* | % | 67 | 63 | 70 | NS |
|  |  | Mean (SD) | 2.5 (6.1) | 4.9 (8.8) | 0.6 (0.7) | NS |
|  | *Gorilla gorilla* | % | 44 | 50 | 40 | NS |
|  |  | Mean (SD) | 1.1 (2.6) | 1.8 (3.8) | 0.5 (1.1) | NS |
|  | *Pan troglodytes* | % | 33 | 25 | 40 | NS |
|  |  | Mean (SD) | 0.5 (1.2) | 0.8 (1.7) | 0.2 (0.3) | NS |
| **Butcher** | *Cercocebus agilis* | % | 44 | 38 | 50 | NS |
|  |  | Mean (SD) | 0.5 (1.1) | 0.7 (1.6) | 0.4 (0.4) | NS |
|  | *Cercopithecus cephus* | % | 72 | 75 | 70 | NS |
|  |  | Mean (SD) | 0.9 (1.1) | 1.2 (1.3) | 0.7 (1) | NS |
|  | *Cercopithecus neglectus* | % | 28 | 25 | 30 | NS |
|  |  | Mean (SD) | 0.3 (0.7) | 0.4 (1.1) | 0.2 (0.3) | NS |
|  | *Cercopithecus nictitans* | % | 94 | 100 | 90 | NS |
|  |  | Mean (SD) | 2.6 (3.7) | 2.7 (3.2) | 2.5 (4.2) | NS |
|  | *Cercopithecus sclateri* | % | 78 | 88 | 70 | NS |
|  |  | Mean (SD) | 1.7 (2.6) | 2 (2.2) | 1.5 (2.9) | NS |
|  | *Colobus guereza* | % | 33 | 50 | 20 | NS |
|  |  | Mean (SD) | 0.4 (0.9) | 0.5 (0.7) | 0.4 (1) | NS |
|  | *Lophocebus albignea* | % | 56 | 50 | 60 | NS |
|  |  | Mean (SD) | 0.9 (2) | 1.5 (2.9) | 0.3 (0.5) | NS |
|  | *Gorilla gorilla* | % | 28 | 38 | 20 | NS |
|  |  | Mean (SD) | 0.2 (0.3) | 0.3 (0.4) | 0.1 (0.2) | NS |
|  | *Pan troglodytes* | % | 11 | 13 | 10 | NS |
|  |  | Mean (SD) | 0.1 (0.3) | 0.1 (0.4) | 0 (0.1) | NS |
| **Cook** | *Cercocebus agilis* | % | 44 | 25 | 60 | NS |
|  |  | Mean (SD) | 0.7 (1.7) | 0.9 (2.5) | 0.5 (0.7) | NS |
|  | *Cercopithecus cephus* | % | 72 | 75 | 70 | NS |
|  |  | Mean (SD) | 1.4 (2.2) | 2.1 (3.1) | 0.8 (1) | NS |
|  | *Cercopithecus neglectus* | % | 28 | 25 | 30 | NS |
|  |  | Mean (SD) | 0.4 (1.1) | 0.6 (1.6) | 0.2 (0.3) | NS |
|  | *Cercopithecus nictitans* | % | 89 | 88 | 90 | NS |
|  |  | Mean (SD) | 3.2 (3.6) | 4.3 (4.6) | 2.3 (2.5) | NS |
|  | *Cercopithecus sclateri* | % | 83 | 88 | 80 | NS |
|  |  | Mean (SD) | 1.7 (1.8) | 2.4 (2.4) | 1.3 (1.1) | NS |
|  | *Colobus guereza* | % | 39 | 38 | 40 | NS |
|  |  | Mean (SD) | 0.3 (0.6) | 0.5 (0.8) | 0.2 (0.3) | NS |
|  | *Lophocebus albignea* | % | 61 | 63 | 60 | NS |
|  |  | Mean (SD) | 1.1 (2.5) | 1.8 (3.7) | 0.5 (0.5) | NS |
|  | *Gorilla gorilla* | % | 22 | 38 | 10 | NS |
|  |  | Mean (SD) | 0.2 (0.6) | 0.4 (0.8) | 0 (0) | NS |
|  | *Pan troglodytes* | % | 11 | 13 | 10 | NS |
|  |  | Mean (SD) | 0.1 (0.4) | 0.2 (0.6) | 0 (0.1) | NS |
| **Consume** | *Cercocebus agilis* | % | 61 | 25 | 90 | * |
|  |  | Mean (SD) | 0.8 (1.7) | 0.9 (2.5) | 0.7 (0.9) | 0.06 |
|  | *Cercopithecus cephus* | % | 78 | 75 | 80 | NS |
|  |  | Mean (SD) | 1.5 (2.2) | 2.1 (3.1) | 1 (1.1) | NS |
|  | *Cercopithecus neglectus* | % | 28 | 25 | 30 | NS |
|  |  | Mean (SD) | 0.4 (1.2) | 0.7 (1.8) | 0.2 (0.3) | NS |
|  | *Cercopithecus nictitans* | % | 94 | 88 | 100 | NS |
|  |  | Mean (SD) | 3.6 (4.1) | 4.4 (4.6) | 3 (3.7) | NS |
|  | *Cercopithecus sclateri* | % | 89 | 88 | 90 | NS |
|  |  | Mean (SD) | 2.2 (2.2) | 2.6 (2.4) | 1.9 (2.1) | NS |
|  | *Colobus guereza* | % | 39 | 38 | 40 | NS |
|  |  | Mean (SD) | 0.4 (0.7) | 0.6 (0.9) | 0.3 (0.5) | NS |
|  | *Lophocebus albignea* | % | 61 | 63 | 60 | NS |
|  |  | Mean (SD) | 1.1 (2.5) | 1.8 (3.7) | 0.5 (0.6) | NS |
|  | *Pan troglodytes* | % | 22 | 13 | 30 | NS |
|  |  | Mean (SD) | 0.2 (0.5) | 0.2 (0.6) | 0.2 (0.4) | NS |
| **All physical** | *Cercocebus agilis* | % | 72 | 50 | 90 | NS |
|  |  | Mean (SD) | 1 (1.7) | 1 (2.4) | 0.9 (1) | NS |
|  | *Cercopithecus cephus* | % | 83 | 88 | 80 | NS |
|  |  | Mean (SD) | 1.6 (2.2) | 2.2 (3) | 1.2 (1.3) | NS |
|  | *Cercopithecus neglectus* | % | 28 | 25 | 30 | NS |
|  |  | Mean (SD) | 0.4 (1.2) | 0.7 (1.8) | 0.2 (0.5) | NS |
|  | *Cercopithecus nictitans* | % | 100 | 100 | 100 | NS |
|  |  | Mean (SD) | 4.6 (5.2) | 4.9 (4.3) | 4.4 (6) | NS |
|  | *Cercopithecus sclateri* | % | 94 | 88 | 100 | NS |
|  |  | Mean (SD) | 3 (3) | 2.8 (2.4) | 3.1 (3.5) | NS |
|  | *Colobus guereza* | % | 50 | 50 | 50 | NS |
|  |  | Mean (SD) | 0.7 (1.3) | 0.7 (1) | 0.7 (1.6) | NS |
|  | *Lophocebus albignea* | % | 67 | 63 | 70 | NS |
|  |  | Mean (SD) | 1.3 (2.5) | 1.9 (3.7) | 0.8 (0.8) | NS |
|  | *Gorilla gorilla* | % | 44 | 38 | 50 |  |
|  |  | Mean (SD) | 0.7 (1.5) | 0.4 (0.8) | 0.9 (1.9) | NS |
|  | *Pan troglodytes* | % | 28 | 13 | 40 | NS |
|  |  | Mean (SD) | 0.3 (0.5) | 0.2 (0.6) | - 1. (0.5) | NS |

1. Scores represent percentage of days with contact over a 10-month period
2. Comparisons of proportion between gender were performed by Fisher exact test, and those for frequencies with Wilcoxon test. p-value < 0.05 (*); < 0.01 (**)NS: Not Significant

**S1_B: Proportion of people in contact with NHP at least once and mean scores of estimated frequency^1^ (questionnaire data) by type of contact for each species and all NHP species combined.**

| **Type of contact** | **Species** |  | **Total (n=449)** | **Women (n=203)** | **Men (n=237)** | **p-value^2^** |
| --- | --- | --- | --- | --- | --- | --- |
| **Injury** | *Cercocebus agilis* | % | 0.4 | 0.5 | 0.4 | NS |
|  |  | Mean (SD) | 0.032 (0.668) | 0.07 (0.992) | 0 (0.007) | NS |
|  | *Cercopithecus cephus* | % | 0.4 | 0 | 0.8 | NS |
|  |  | Mean (SD) | 0 (0.007) | 0 (0) | 0.001 (0.009) | NS |
|  | *Cercopithecus neglectus* | % | 0 | 0 | 0 | / |
|  |  | Mean (SD) | / | / | / | / |
|  | *Cercopithecus nictitans* | % | 2 | 1.5 | 2.5 | NS |
|  |  | Mean (SD) | 0.01 (0.144) | 0.001 (0.012) | 0.017 (0.199) | NS |
|  | *Cercopithecus sclateri* | % | 0 | 0 | 0 | / |
|  |  | Mean (SD) | / | / | / | / |
|  | *Colobus guereza* | % | 0.2 | 0 | 0.4 | NS |
|  |  | Mean (SD) | 0 (0.005) | 0 (0) | 0 (0.007) | NS |
|  | *Lophocebus albigena* | % | 0.4 | 0.5 | 0.4 | NS |
|  |  | Mean (SD) | 0.001 (0.015) | 0.002 (0.021) | 0 (0.007) | NS |
|  | *Gorilla gorilla* | % | 2.9 | 0 | 5.1 | ** |
|  |  | Mean (SD) | 0.003 (0.017) | 0 (0) | 0.005 (0.022) | ** |
|  | *Pan troglodytes* | % | 0.7 | 0 | 1.3 | NS |
|  |  | Mean (SD) | 0.001 (0.016) | 0 (0) | 0.002 (0.022) | NS |
| **Hunt** | *Cercocebus agilis* | % | 31 | 3.9 | 54 | *** |
|  |  | Mean (SD) | 0.666 (5.109) | 0.033 (0.298) | 0.737 (2.467) | *** |
|  | *Cercopithecus cephus* | % | 33.9 | 4.9 | 57.8 | *** |
|  |  | Mean (SD) | 2.592 (12.724) | 0.078 (0.983) | 4.433 (16.086) | *** |
|  | *Cercopithecus neglectus* | % | 19.4 | 1.5 | 33.3 | *** |
|  |  | Mean (SD) | 0.135 (0.807) | 0.001 (0.012) | 0.239 (1.083) | ** |
|  | *Cercopithecus nictitans* | % | 42.3 | 5.4 | 73 | *** |
|  |  | Mean (SD) | 2.184 (10.89) | 0.023 (0.214) | 3.961 (14.699) | *** |
|  | *Cercopithecus sclateri* | % | 27.6 | 2 | 48.5 | *** |
|  |  | Mean (SD) | 1.86 (9.885) | 0.032 (0.298) | 3.38 (13.412) | *** |
|  | *Colobus guereza* | % | 21.6 | 2 | 37.6 | *** |
|  |  | Mean (SD) | 0.449 (4.893) | 0.033 (0.299) | 0.809 (6.71) | NS |
|  | *Lophocebus albigena* | % | 25.4 | 2 | 44.7 | *** |
|  |  | Mean (SD) | 0.403 (1.64) | 0.016 (0.211) | 0.707 (2.177) | *** |
|  | *Gorilla gorilla* | % | 19.4 | 1 | 34.6 | *** |
|  |  | Mean (SD) | 0.072 (0.351) | 0.003 (0.03) | 0.132 (0.474) | *** |
|  | *Pan troglodytes* | % | 14.7 | 1 | 25.3 | *** |
|  |  | Mean (SD) | 0.081 (0.722) | 0.003 (0.03) | 0.149 (0.989) | * |
| **Buy/Sell** | *Cercocebus agilis* | % | 47.2 | 38.9 | 55.7 | *** |
|  |  | Mean (SD) | 3.844 (15.017) | 3.801 (15.687) | 4.011 (14.691) | NS |
|  | *Cercopithecus cephus* | % | 64.8 | 58.1 | 71.3 | ** |
|  |  | Mean (SD) | 10.015 (24.375) | 8.64 (21.64) | 11.488 (26.809) | NS |
|  | *Cercopithecus neglectus* | % | 25.6 | 18.2 | 32.1 | *** |
|  |  | Mean (SD) | 1.818 (9.88) | 2.226 (12.414) | 1.47 (7.207) | NS |
|  | *Cercopithecus nictitans* | % | 71.5 | 67.5 | 75.5 | * |
|  |  | Mean (SD) | 11.286 (24.932) | 9.867 (23.289) | 12.803 (26.592) | NS |
|  | *Cercopithecus sclateri* | % | 61 | 52.7 | 68.8 | *** |
|  |  | Mean (SD) | 9.255 (24.153) | 8.227 (23.667) | 10.413 (24.924) | NS |
|  | *Colobus guereza* | % | 41.2 | 35 | 47.7 | * |
|  |  | Mean (SD) | 2.106 (9.887) | 0.818 (2.508) | 3.278 (13.293) | * |
|  | *Lophocebus albigena* | % | 51.2 | 43.3 | 58.6 | ** |
|  |  | Mean (SD) | 4.747 (16.411) | 4.211 (15.782) | 5.307 (17.19) | NS |
|  | *Gorilla gorilla* | % | 44.5 | 40.4 | 49.4 | * |
|  |  | Mean (SD) | 1.335 (7.121) | 0.714 (2.131) | 1.921 (9.599) | NS |
|  | *Pan troglodytes* | % | 28.1 | 19.7 | 35.9 | *** |
|  |  | Mean (SD) | 0.411 (1.642) | 0.219 (1.14) | 0.59 (1.984) | * |
| **Butcher** | *Cercocebus agilis* | % | 63.9 | 55.2 | 70.9 | *** |
|  |  | Mean (SD) | 5.262 (16.399) | 5.376 (17.175) | 5.255 (15.997) | NS |
|  | *Cercopithecus cephus* | % | 80.4 | 75.9 | 84.8 | * |
|  |  | Mean (SD) | 13.702 (27.326) | 12.067 (24.719) | 15.416 (29.712) | NS |
|  | *Cercopithecus neglectus* | % | 80.4 | 75.9 | 84.8 | *** |
|  |  | Mean (SD) | 2.12 (9.954) | 1.891 (10.279) | 2.269 (9.79) | NS |
|  | *Cercopithecus nictitans* | % | 85.5 | 78.8 | 91.1 | *** |
|  |  | Mean (SD) | 15.451 (29.249) | 15.804 (29.991) | 15.44 (29.099) | NS |
|  | *Cercopithecus sclateri* | % | 74.8 | 67.5 | 81.4 | ** |
|  |  | Mean (SD) | 11.832 (25.968) | 9.295 (22.487) | 13.836 (28.272) | 0.07 |
|  | *Colobus guereza* | % | 58.4 | 49.3 | 65.8 | *** |
|  |  | Mean (SD) | 3.6 (12.848) | 2.383 (8.035) | 4.741 (15.965) | 0.06 |
|  | *Lophocebus albigena* | % | 66.4 | 56.7 | 74.3 | *** |
|  |  | Mean (SD) | 6.423 (19.212) | 5.187 (17.149) | 7.503 (21.076) | NS |
|  | *Gorilla gorilla* | % | 65.7 | 56.7 | 73.4 | *** |
|  |  | Mean (SD) | 1.632 (7.267) | 1.481 (7.491) | 1.803 (7.201) | NS |
|  | *Pan troglodytes* | % | 49.9 | 39.4 | 58.6 | *** |
|  |  | Mean (SD) | 0.714 (2.206) | 0.45 (1.799) | 0.902 (2.364) | * |
| **Cook** | *Cercocebus agilis* | % | 53.2 | 55.2 | 51.5 | NS |
|  |  | Mean (SD) | 3.112 (11.959) | 4.083 (14.348) | 2.306 (9.639) | NS |
|  | *Cercopithecus cephus* | % | 73.3 | 79.3 | 68.8 | * |
|  |  | Mean (SD) | 10.001 (23.163) | 9.678 (21.5) | 10.532 (24.86) | NS |
|  | *Cercopithecus neglectus* | % | 38.5 | 34.5 | 41.4 | NS |
|  |  | Mean (SD) | 1.887 (9.952) | 2.413 (12.587) | 1.433 (7.185) | NS |
|  | *Cercopithecus nictitans* | % | 77.3 | 80.8 | 75.1 | NS |
|  |  | Mean (SD) | 10.331 (23.092) | 13.456 (27.708) | 7.863 (18.308) | * |
|  | *Cercopithecus sclateri* | % | 66.8 | 69.5 | 64.6 | NS |
|  |  | Mean (SD) | 8.623 (22.168) | 8.653 (22.757) | 7.909 (20.301) | NS |
|  | *Colobus guereza* | % | 50.8 | 50.7 | 51.1 | NS |
|  |  | Mean (SD) | 1.402 (5.532) | 1.07 (2.695) | 1.731 (7.181) | NS |
|  | *Lophocebus albigena* | % | 59.5 | 59.6 | 59.1 | NS |
|  |  | Mean (SD) | 4.521 (15.808) | 4.451 (14.411) | 4.67 (17.165) | NS |
|  | *Gorilla gorilla* | % | 65.5 | 68 | 63.7 | NS |
|  |  | Mean (SD) | 1.024 (2.704) | 0.988 (2.514) | 1.075 (2.898) | NS |
|  | *Pan troglodytes* | % | 46.5 | 44.3 | 48.5 | NS |
|  |  | Mean (SD) | 0.614 (2.179) | 0.518 (2.046) | 0.656 (2.15) | NS |
| **Consume** | *Cercocebus agilis* | % | 65.3 | 54.7 | 74.7 | *** |
|  |  | Mean (SD) | 3.57 (12.101) | 4.122 (14.415) | 3.13 (9.9) | NS |
|  | *Cercopithecus cephus* | % | 79.7 | 75.9 | 83.5 | * |
|  |  | Mean (SD) | 12.593 (26.316) | 9.797 (21.552) | 15.222 (29.925) | * |
|  | *Cercopithecus neglectus* | % | 48.1 | 35 | 59.1 | *** |
|  |  | Mean (SD) | 2.006 (10.042) | 2.438 (12.651) | 1.631 (7.303) | NS |
|  | *Cercopithecus nictitans* | % | 81.5 | 73.9 | 88.2 | *** |
|  |  | Mean (SD) | 15.469 (29.385) | 14.195 (28.469) | 16.909 (30.555) | NS |
|  | *Cercopithecus sclateri* | % | 75.7 | 68.5 | 81.9 | ** |
|  |  | Mean (SD) | 11.052 (25.215) | 9.84 (24.688) | 12.281 (26.022) | NS |
|  | *Colobus guereza* | % | 59.5 | 51.2 | 67.1 | *** |
|  |  | Mean (SD) | 2.156 (8.805) | 1.39 (3.368) | 2.879 (11.709) | NS |
|  | *Lophocebus albigena* | % | 66.8 | 53.2 | 78.1 | *** |
|  |  | Mean (SD) | 5.652 (17.716) | 4.262 (14.482) | 6.931 (20.242) | NS |
|  | *Gorilla gorilla* | % | 70.2 | 61.6 | 77.6 | *** |
|  |  | Mean (SD) | 1.698 (7.297) | 0.953 (2.517) | 2.387 (9.74) | * |
|  | *Pan troglodytes* | % | 53 | 41.9 | 62.4 | *** |
|  |  | Mean (SD) | 0.692 (2.203) | 0.457 (1.813) | 0.855 (2.353) | 0.05 |
| **All physical** | *Cercocebus agilis* | % | 73.1 | 64 | 80.6 | NS |
|  |  | Mean (SD) | 7.031 (19.113) | 6.799 (19.537) | 6.884 (18.126) | NS |
|  | *Cercopithecus cephus* | % | 87.8 | 83.7 | 91.1 | NS |
|  |  | Mean (SD) | 19.092 (32.323) | 15.424 (27.9) | 22.366 (35.486) | * |
|  | *Cercopithecus neglectus* | % | 53.9 | 38.9 | 66.2 | *** |
|  |  | Mean (SD) | 2.774 (11.918) | 2.997 (14.154) | 2.568 (9.837) | NS |
|  | *Cercopithecus nictitans* | % | 91.1 | 85.7 | 95.8 | NS |
|  |  | Mean (SD) | 22.594 (35.163) | 19.979 (33.263) | 25.405 (37.1) | NS |
|  | *Cercopithecus sclateri* | % | 83.5 | 76.4 | 89.5 | NS |
|  |  | Mean (SD) | 16.096 (30.275) | 11.765 (25.601) | 19.428 (32.974) | ** |
|  | *Colobus guereza* | % | 69.7 | 59.6 | 78.5 | NS |
|  |  | Mean (SD) | 4.375 (13.659) | 2.95 (8.234) | 5.721 (17.09) | * |
|  | *Lophocebus albigena* | % | 77.5 | 67.5 | 85.7 | * |
|  |  | Mean (SD) | 8.147 (20.992) | 6.819 (19.512) | 9.376 (22.496) | NS |
|  | *Gorilla gorilla* | % | 80.4 | 74.4 | 85.7 | NS |
|  |  | Mean (SD) | 2.531 (9.871) | 1.81 (7.531) | 3.229 (11.631) | NS |
|  | *Pan troglodytes* | % | 61.7 | 52.2 | 69.6 | ** |
|  |  | Mean (SD) | 1.043 (2.76) | 0.771 (2.46) | 1.253 (2.904) | 0.06 |

^1^ Estimated frequencies were calculated as follow: 0=never. 0.1=more than one year. 0.3=during the previous year. 3=during the previous month. 14=during the previous week and 100=yesterday.

^2^ Comparisons of proportion between gender were performed by Fisher exact test, and those for frequencies with Student t test. NS: Not Significant. p-value < 0.05 (*); <0.01 (**); < 0.001 (***). Differences of sample size between “Total” and “Women+Men” are due to 9 missing value for gender information.
